# Indistinguishability as nonlocality constraint

**DOI:** 10.1038/s41598-018-24489-7

**Published:** 2018-04-17

**Authors:** Cássio Sozinho Amorim

**Affiliations:** Nagoya University, Department of Applied Physics, Nagoya, 464-8603 Japan

## Abstract

A physical explanation for quantum bounds to nonlocality (Tsirelson’s bound) is a fundamental problem in quantum theory, for it is known that no-signaling alone fails to reproduce this limit. Here, information indistinguishability is presented as the indistinguishability of qubits or more general bits, and it suggests an answer to the nonlocality conundrum, ultimately placing it as the origin to quantum limits. Indistinguishability is also connected to exclusivity principle, and it is shown that indistinguishability leads to quantum correlation bounds. This suggests indistinguishability be as fundamental as non-locality and relativistic causality for nonlocal realism.

## Introduction

Quantum theory: although arcane lore for the laymen, for physicists it constitutes a solid foundation for investigating microscopic phenomena amidst bewilderment that challenges common sense continuously. Entanglement, the quantum correlation between pieces of a system that is not separable into a direct product of individual particles’ states, is central to such bewilderment. Here, we denote by “quantum correlations” functions of the expectation value of two or more two-level systems or quantum bits (qubits), like spin-half particles. The correlations underlying quantum theory, such as those pointed in the EPR paradox^1^, are examples of entanglement’s counterintuitive consequences. These correlations are nonlocal, and they are guaranteed by proofs that forbid the theory to become deterministic through local hidden variables, barring away local realism, i.e., the assumption of hidden variables associated with specific local components^2–6^. A well-known correlation function between two qubits is the Clauser-Horne-Shimony-Holt (CHSH) correlation^3^, given by

1 $$\langle {S}_{2}\rangle =\langle {a}_{1}^{0}{a}_{2}^{0}\rangle +\langle {a}_{1}^{0}{a}_{2}^{1}\rangle +\langle {a}_{1}^{1}{a}_{2}^{0}\rangle -\langle {a}_{1}^{1}{a}_{1}^{1}\rangle ,$$

where ${a}_{i}^{k}$ are measurement operators acting on qubits with outcome ±1, subscripts labeling the qubit acted upon, and superscripts indicating one of two different bases. 〈〉 stands for expectation value. More recently, two groups have independently generalized CHSH correlation for *n*-particle correlations as a polynomial function that can be expressed recursively as

2 $${S}_{n}={S}_{n-1}{a}_{n}^{0}-{\bar{S}}_{n-1}{a}_{n}^{1},$$

starting from *S*_2_, where ${\bar{S}}_{n}$ is obtained by swapping 0/1 bases of measurement^7,8^. These correlation functions are limited by the *n*-body Tsirelson’s bound

3 $$|\langle {S}_{n}\rangle |\le {2}^{n-1}\sqrt{2}\mathrm{.}$$

In fact, CHSH correlation first appeared as a Bell inequality^2^ for two particles considering the limits of quantum correlations with hidden variables defining outcomes, and later Tsirelson showed that the quantum mechanical limits are greater by a factor of $\sqrt{2}$^9^. This nonlocal realism has also been confirmed in experiments^10–12^.

Nevertheless, Popescu and Rohrlich further highlighted that one could have even stronger correlations while preserving the principle that no information can be transmitted faster than light or “no-signaling” for short^13^. They showed that the algebraic limit for CHSH inequality (|〈*S*_2_〉| ≤ 4), where every term assumes value one, can be theoretically achieved without any superluminal transmission. This begs the question: besides no-signaling, is there any other fundamental limit to correlations in nature? This matter has been explored in recent years through different perspectives, namely information causality^14^, macroscopic locality^15^, and exclusivity principle (E principle)^16^. Information causality provides an information theoretical approach to the problem, limiting the amount of information inferred about a distant party by local operations to the number of classical bits transmitted. Macroscopic locality imposes constraints to correlations generated by any no-signaling box (NS-box) in order to recover classical world. E principle follows a graph theoretical approach to recover Tsirelson’s bound in general probabilistic theories (GPT). In this paper, we try a new principle: information indistinguishability.

The indistinguishability of identical particles has been taken into consideration since Gibbs paradox in statistical mechanics, where the entropy of an ideal gas of particles does not become extensive if one ignores particle indistinguishability. In quantum mechanics, entanglement between identical particles has been investigated by different approaches^17–20^. While these approaches discuss the entanglement formation related to indistinguishability of bosons and fermions, some aspects may not be so clear when one considers a more general situation involving, for example, anyons or states in GPTs. This motivates us to consider indistinguishability not only between identical particles (bosons and fermions), but information units themselves (i.e., qubits and their possible generalizations).

In this paper, by extending the notion of indistinguishability to information units, namely bits, we show that information indistinguishability may explain Tsirelson’s bound^9^, including its generalized form for *n* qubits^7,8^. We first revisit quantum theory to redefine indistinguishability and qubits, later using it to define information indistinguishability. Then, we show that information indistinguishability leads to entanglement generation between qubits and that the limit for such entanglement coincides with Tsirelson’s bound. Finally, we generalize our qubits to general bits (gbits) in GPTs following Chiribella and Yuan^21^, provide proof of Tsirelson’s bound for *n* gbits, and provide a connection with E principle.

## Qubit Distinction and Information Indistinguishability

Starting from quantum theory, let us first clarify the word “indistinguishability”. Since one cannot assume indistinguishability to always hold between two particles, which can even bear different statistics (viz. bosonic/fermionic/fractional), we consider two qubits that may be completely distinct. Henceforth, the word “distinct” shall be used to indicate particles (in first quantization) or modes (in second quantization) that can be unambiguously characterized as different by arbitrary means, like a proton and an electron by their charge and mass, or two particles sufficiently far apart. On the other hand, we resort to the word “distinguishable” to imply the possibility of unambiguous identification of particles or states with finite probability^22–24^. We say two distinct qubits are rendered “indistinguishable” when their internal states are mixed (by coupling or scattering), and their information cannot be uniquely tracked. For example, a proton and an electron: we cannot independently track their spins in the ground state of a hydrogen atom without breaking some symmetry to attach each spin unambiguously to each particle; they are distinct, but their spins become indistinguishable. If we can uniquely identify states with probability 1, we say they are “perfectly distinguishable”.

For handling indistinguishability, we shall first focus on quantum states in a Hilbert space under some symmetry and later relate it to qubit indistinguishability. We consider some symmetry ${\mathfrak{M}}$ mutually mapping indistinguishable states of the (two-particle) Hilbert space ${\mathcal H}$,

4 $${\mathfrak{M}}: {\mathcal H} \to  {\mathcal H} \mathrm{.}$$

This symmetry between states also correlates qubits composing such states, amounting to some symmetry between qubits themselves. That is, being states related as ${\mathfrak{M}}|\psi \rangle =|\psi ^{\prime} \rangle$ indistinguishable, two qubits assuming states |*ψ*〉 and |*ψ*′〉 are also said indistinguishable. Therefore, we may conversely discuss qubit or information indistinguishability as well as state indistinguishability (or symmetry). Because ${\mathfrak{M}}$ defines indistinguishability between qubits by mapping states ${\mathfrak{M}}|\psi \rangle \to |\psi ^{\prime} \rangle$, its eigenstates must be symmetric superpositions of indistinguishable states. If we assume indistinguishability to generate correlations, we shall thus expect maximum correlations to be encoded in these eigenstates.

**Figure 1:**
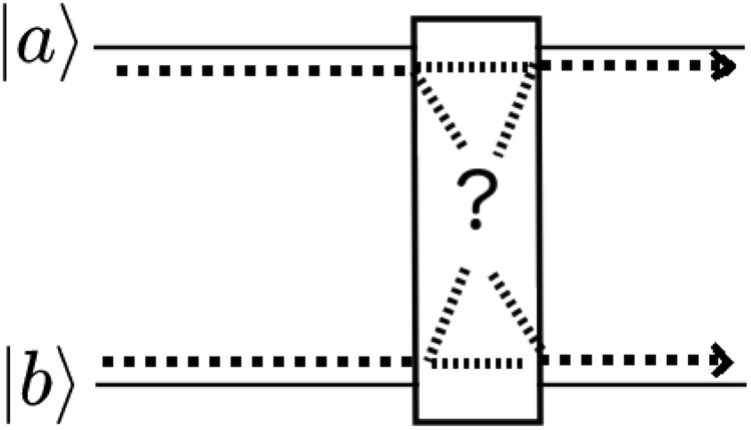
Two qubits |*a*〉 and |*b*〉 (with reference input implicitly identified by their lines) going through an entangling transformation. Given only the output, and the knowledge of the existence of a two-qubit transformation, it is impossible to tell which path the information follows.

Nevertheless, we know that we often can distinguish states when such symmetry does not exist. We thus need to clarify when such symmetry holds or not and a way to identify whether we have distinguishable states or not. Regarding the first, as indistinguishability (or its equivalent symmetry) is assumed to be the source of entanglement, it takes place during generation of entanglement. Once the symmetry has been imposed on the whole state, each qubit can later propagate independently. Therefore, one may consider the input of distinguishable uncorrelated qubits that undergo a symmetrization process through a region with symmetry. In this region, it should be impossible to uniquely identify each qubit’s “path” through the symmetrization process. Later, leaving the symmetric region, qubits may once again become distinguishable following independent paths, though now correlated (see Fig. 1).

To identify distinguishable states, it suffices to note that before and after symmetrization, qubits may have their states defined by some physical background that may tag each qubit, but become inaccessible in the symmetric region. Take, for instance, the original EPR paradox: two photons coming from opposite directions collide to generate an electron-positron pair in a singlet state. Before and after the scattering process, qubits (spin polarization) can be identified by left/right photon or electron/positron spin, but not during the scattering process. Therefore, we make this label explicit, rewriting the bases as |*r*_1_, *q*_1_〉 |*r*_2_, *q*_2_〉, where *r* is a label that physically identifies the qubit and provides a reference against which a qubit encoding information *q* is defined. When the two qubits become indistinguishable, we consider that they become each other’s reference, losing references *r*. For example, one could write |*e*^−^, 0〉 |*e*^+^, 1〉 for an electron/positron pair far apart. If one considers their creation or annihilation process, a singlet state $\mathrm{(|01}\rangle -\mathrm{|10}\rangle )/\sqrt{2}$ where the spins become their mutual reference and only an overall relative information exists (antiparallel) is obtained as the product of particle exchange symmetry, without identifying a spin with one particle. Note that such label may be recovered when particles leave symmetric regime, but we shall consider mainly the “indistinguishing process”. We shall see next how can we handle this transition from distinguishable to indistinguishable.

## Excluding Physical Background

To exclude the physical background information in *r*, we adapt the procedure by Lo Franco and Compagno and Sciara *et al*.^17,18^, introducing a state symmetrization in partial inner products. Lo Franco and Compagno define a non-separable symmetric external product of one-particle states $|{\psi }_{1},{\psi }_{2}\rangle \,:\,=|{\psi }_{1}\rangle \times |{\psi }_{2}\rangle$ on one-particle basis ${ {\mathcal B} }^{\mathrm{(1)}}=\{|{\psi }_{k}\rangle |k\in {\mathbb{N}}\}$, so that ${\langle {\psi }_{1},{\psi }_{2}|=(|{\psi }_{1},{\psi }_{2}\rangle )}^{\dagger }=\langle {\psi }_{2}|\times \langle {\psi }_{1}|$ and |*ψ*_1_〉 × |*ψ*_2_〉 = *η*|*ψ*_2_〉 × |*ψ*_1_〉, where *η* = ±1 is a factor accounting for the statistics of the particles^17^. With this, they define a symmetric (unnormalized) inner product:

5 $$\langle \xi |\psi ,\varphi \rangle =\langle \xi |\psi \rangle |\varphi \rangle +\eta \langle \xi |\varphi \rangle |\psi \rangle ,$$

with $|\xi \rangle \in { {\mathcal B} }^{\mathrm{(1)}}$. In this inner product, the symmetry imposed is that of particle permutation, according to bosonic of fermionic statistics. This product works as a projection of a two-body state of indistinguishable particles onto a single-body state of them.

We need to change this product to perform two tasks: (a) impose symmetries ${\mathfrak{M}}$ not restricted to particle permutation, that may also account for qubit indistinguishability and (b) impose such symmetry by dropping their distinguishing information (background reference) and establishing the components as mutual references. For (a), we just need to redefine the symmetric external product with our symmetry ${\mathfrak{M}}$, such that the relation ${\mathfrak{M}}|{\psi }_{1},{\psi }_{2}\rangle =|{\psi ^{\prime} }_{1},{\psi ^{\prime} }_{2}\rangle$. Equation (5) is derived by Lo Franco and Compagno^17^ from the action of single-paticle operators, specifically single-particle projector, on the symmetric state defined. In their framework of particle indistinguishability, a one-particle operator *A*^(1)^ must act as *A*^(1)^|*ψ*_1_, *ψ*_2_〉 = |*A*^(1)^*ψ*_1_, *ψ*_2_〉 + *η*|*ψ*_1_, *A*^(1)^*ψ*_2_〉. For symmetries other than particle exchange, the one-particle operator acts like *A*^(1)^|*ψ*_1_, *ψ*_2_〉 = |*A*^(1)^*ψ*_1_, *ψ*_2_〉 + |*A*^(1)^${\psi ^{\prime} }_{1}$, ${\psi ^{\prime} }_{2}$〉 or *A*^(1)^|*ψ*_1_, *ψ*_2_〉 = |*ψ*_1_, *A*^(1)^*ψ*_2_〉 + |${\psi ^{\prime} }_{1}$, *A*^(1)^${\psi ^{\prime} }_{2}$〉. Consequently, the action of a projector |*ξ*〉 〈*ξ*| on a state |*ψ*, *ϕ*〉 can be straightforwardly identified as $|\xi \rangle \times (\langle \xi |\psi \rangle |\varphi \rangle +\langle \xi |\psi ^{\prime} \rangle |\varphi ^{\prime} \rangle )$, whence we can generalize equation (5) as

6 $$\langle \xi |\psi ,\varphi \rangle =\langle \xi |\psi \rangle |\varphi \rangle +\langle \xi |\psi ^{\prime} \rangle |\varphi ^{\prime} \rangle \mathrm{.}$$

If ${\mathfrak{M}}$ is particle exchange symmetry, equation (5) is recovered by the relations |*ψ*′〉 = *η*|*ϕ*〉 and |*ϕ*′〉 = |*ψ*〉. Furthermore, we need to reconsider equation (6) to accomplish (b). For this, we treat the physical references as quantum states where the qubits are defined and rewrite equation (6) as

7 $$\langle {r}_{1},{r}_{2}|{r}_{1},\psi ;{r}_{2}\varphi \rangle =\langle {r}_{1}|{r}_{1},\psi \rangle \langle {r}_{2}|{r}_{2},\varphi \rangle +\langle {r}_{1}|{r}_{1},\psi ^{\prime} \rangle \langle {r}_{2}|{r}_{2},\varphi ^{\prime} \rangle \mathrm{.}$$

This will lead to a state $|\psi ,\varphi \rangle +\tilde{\eta }|\psi ^{\prime} ,\varphi ^{\prime} \rangle$, with $|\tilde{\eta }|=1$ according to symmetry ${\mathfrak{M}}$. Essentially, ${\mathfrak{M}}$ destroys the reference information *r* and imposes some symmetry, like particle exchange or time reversal, in this process. For example, if we start from an electron/positron pair of opposite spins and ${\mathfrak{M}}$ account for particle exchange while annihilating the particles, we would have

8 $$\begin{array}{rcl}\langle {e}^{-},{e}^{+}|{e}^{-},\,\mathrm{0;}\,{e}^{+}1\rangle  & = & \langle {e}^{-}|{e}^{-},\,0\rangle \langle {e}^{+}|{e}^{+},\,1\rangle \\  &  & +\,\eta \langle {e}^{-}|{e}^{-},\,1\rangle \langle {e}^{+}|{e}^{+},\,0\rangle \mathrm{.}\\  & = & \mathrm{|0},1\rangle -\mathrm{|1},0\rangle \mathrm{.}\end{array}$$

Similarly, we could consider the imposition of time reversal symmetry on a superconducting (SC) qubit and an NV-center (NV) coupled together, starting from the ground state:

9 $$\begin{array}{rcl}\langle {\rm{SC}},{\rm{NV}}|{\rm{SC}},\,0;{\rm{NV}},\,0\rangle  & = & \langle {\rm{SC}}|{\rm{SC}},\,0\rangle \langle {\rm{NV}}|{\rm{NV}},\,0\rangle \\  &  & +\,\tilde{\eta }\langle {\rm{SC}}|{\rm{SC}},\,1\rangle \langle {\rm{NV}}|{\rm{NV}},\,1\rangle ,\\  & = & \mathrm{|0},0\rangle +\mathrm{|1},1\rangle ,\end{array}$$

where $\tilde{\eta }$ account for the general phase gained in the |0, 0〉 → |1, 1〉 transformation, which we assume to be 1 since it is not important for our purposes.

This generalization also makes states $\mathrm{|0;}\,\overrightarrow{s}\rangle$ and $\mathrm{|1};\overrightarrow{\bar{s}}\rangle$ become indistinguishable under certain symmetries, where $\overrightarrow{s}$ indicates a general superposition for the second qubit, viz. $|\overrightarrow{s}\rangle =\,\cos \,\frac{\theta }{2}\mathrm{|0}\rangle +{e}^{i\phi }\,\sin \,\frac{\theta }{2}\mathrm{|1}\rangle$, and $\overrightarrow{\bar{s}}$ is obtained by exchanging |0〉 and |1〉. We therefore realize a state of two qubits where only their relative value has meaning by imposing a symmetry where states of same parity are equivalent. This shall become clearer in the next section, once we look at their Schmidt decomposition (SD).

## Schmidt Decomposition for Indistinguishable Qubits

It is possible to define an SD following Sciara *et al*.^18^ and show that the Schmidt rank obtained is greater than 1 for qubits under indistinguishable condition. Later, we shall see that the upper bound for such entanglement coincides with Tsirelson’s bound.

To perform an SD, one must look into the density operator *ρ* of a given state and (i) reduce it to a single particle (qubit) operator. For this, equation (5) is employed to project the density operator onto single particle basis. Then, (ii) by diagonalizing the reduced density matrix *ρ*^(1)^, the retrieved eigenvectors can be adequately put together to form Schmidt bases, balanced by the singular values (i.e., the square root of eigenvalues) for each eigenstate.

The following example of two bosonic qubits has been worked by Sciara *et al*.^18^. Two qubits in a state $|{\rm{\Phi }}\rangle =\mathrm{|0},\overrightarrow{s}\rangle$ and density matrix *ρ* = |Φ〉 〈Φ|. The single particle density matrix is obtained by performing a partial trace on *ρ* with inner products defined as equation (5), leading to

10 $${\rho }^{\mathrm{(1)}}=\frac{1}{2N}(\begin{array}{cc}a & c\\ {c}^{\ast } & b\end{array}),$$

with $a=4\,{\cos }^{2}\frac{\theta }{2}+{\sin }^{2}\frac{\theta }{2}$, $b={\sin }^{2}\frac{\theta }{2}$, $c={e}^{i\phi }\,\sin \,\theta$, and $N=1+{\cos }^{2}\frac{\theta }{2}$. Schmidt bases can then be derived simply by diagonalizing the reduced density matrix, which in this example gives two eigenvalues

11 $${\lambda }_{0}=\frac{2}{N}\,{\cos }^{4}\,\frac{\theta }{4}\,{\rm{and}}\,{\lambda }_{1}=\frac{2}{N}\,{\sin }^{4}\,\frac{\theta }{4}$$

that are the square of the singular values giving the weight of the respective eigenstates

12 $$\begin{array}{rcl}|\tilde{0}\rangle  & = & \cos \,\frac{\theta }{4}\mathrm{|0}\rangle +\,\sin \,\frac{\theta }{4}\mathrm{|1}\rangle ,\\ |\tilde{1}\rangle  & = & -\sin \,\frac{\theta }{4}\mathrm{|0}\rangle +\,\cos \,\frac{\theta }{4}\mathrm{|1}\rangle \mathrm{.}\end{array}$$

The state can then be written in Schmidt bases as

13 $$|{\rm{\Phi }}\rangle =(\sqrt{{\lambda }_{0}}|\tilde{0},\tilde{0}\rangle +\sqrt{{\lambda }_{1}}|\tilde{1},\tilde{1}\rangle )\mathrm{.}$$

Notice that before the SD, we have one qubit working as the reference, in this case, set to zero, and one general superposition for the coding bit. This state is also equivalent, in our setup, to the state $\mathrm{|1},\overrightarrow{\bar{s}}\rangle$, and some degree of entanglement is expected. Sciara *et al*.^18^ also study the von Neumann entropy $S=-\,{\sum }_{i}\,{\lambda }_{i}\,\mathrm{log}\,{\lambda }_{i}$^25,26^ of this system, which in the extreme case of *θ* = *π* gives exactly one bit of entanglement entropy. This is expected and can be understood as the product of indistinguishability by exchange symmetry between bases |0, 1〉 and |1, 0〉.

Similarly, we can go beyond exchange symmetry of particles explored in previous works and consider other kinds of symmetries. For example, equation (6) and time reversal symmetry that flips 0 ↔ 1 to impose indistinguishability between these bases, |0, 1〉 and |1, 0〉, as well as between |0, 0〉 and |1, 1〉. In this case, our symmetry transformation reads $|\psi ^{\prime} \rangle ={\mathfrak{M}}|\psi \rangle =|\bar{\psi }\rangle$ (i.e. Not *ψ*) and equally to *ϕ*, flipping 0 s and 1 s. Also, note that time reversal manifests itself by changing |0〉 → |1〉 and $\mathrm{|1}\rangle \to \tilde{\eta }\mathrm{|0}\rangle$, with $\tilde{\eta }=\pm 1$ depending on the system considered (i.e., whether time reversal operator squares to 1 or −1). Here, we shall take the case of $\tilde{\eta }=+\,1$. Therefore, applied to state |Φ〉, we have

14 $$\begin{array}{lllllll}\langle \mathrm{0|}{\rm{\Phi }}\rangle  & = & \langle \mathrm{0|}\cdot \mathrm{|0},\overrightarrow{s}\rangle  & = & \langle \mathrm{0|0}\rangle |\overrightarrow{s}\rangle +\langle \mathrm{0|1}\rangle |\overrightarrow{\bar{s}}\rangle  & = & |\overrightarrow{s}\rangle ,\\ \langle \mathrm{1|}{\rm{\Phi }}\rangle  & = & \langle \mathrm{1|}\cdot \mathrm{|0},\overrightarrow{s}\rangle  & = & \langle \mathrm{1|0}\rangle |\overrightarrow{s}\rangle +\langle \mathrm{1|1}\rangle |\overrightarrow{\bar{s}}\rangle  & = & |\overrightarrow{\bar{s}}\rangle \mathrm{.}\end{array}$$

**Figure 2:**
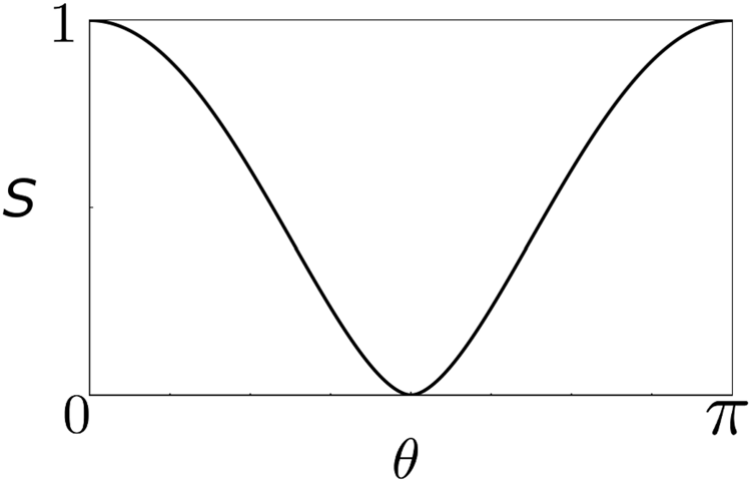
Entanglement entropy (von Neumann) $S=-\,{\lambda }_{+}\,{\mathrm{log}}_{2}\,{\lambda }_{+}-{\lambda }_{-}\,{\mathrm{log}}_{2}\,{\lambda }_{-}$ calculated based on reduced density matrix *ρ*^(1)^ of state |Φ〉 (a pair of qubits with relative inclination *θ*) under time reversal symmetry. Parallel (*θ* = 0) and anti-parallel (*θ* = 1) cases show maximum entanglement.

Hence, under time-reversal symmetry, we obtain the reduced density matrix

15 $${\rho }^{\mathrm{(1)}}=\frac{1}{2}(\begin{array}{cc}1 & \sin \,\theta \\ \sin \,\theta  & 1\end{array}),$$

if one ignores phase factor *e*^*iφ*^, and eigenvalues

16 $${\lambda }_{\pm }=\frac{1\pm \,\sin \,\theta }{2},$$

and corresponding eigenvectors

17 $$|\,\pm \,\rangle =\frac{\mathrm{|0}\rangle \pm \mathrm{|1}\rangle }{\sqrt{2}},$$

leading to the expression on Schmidt bases as

18 $$|{\rm{\Phi }}\rangle =\sqrt{{\lambda }_{+}}|++\rangle +\sqrt{{\lambda }_{-}}|--\rangle ,$$

for the bosonic case. One can calculate von Neumann entanglement entropy from these eigenvalues, $S=-\,{\lambda }_{+}\,{\mathrm{log}}_{2}\,{\lambda }_{+}-{\lambda }_{-}\,{\mathrm{log}}_{2}\,{\lambda }_{-}$, obtaining maximum entanglement for *θ* = 0 (parallel states) and *θ* = *π* (anti-parallel states), as shown in Fig. 2.

Note that this kind of symmetry has the same effect as particle exchange for antiparallel states, but also connects parallel basis, making it easier to deal with for indistinguishability of same parity bases in general. These approaches universalize standard SD and permits to discuss indistinguishable states (see also Appendix 2).

## Schmidt Rank under Indistinguishability

The non-zero von Neumann entropy is indicative of entanglement between indistinguishable bases *per se*, but one can go further and look at Schmidt’s rank for states perfectly indistinguishable, i.e., occupying only indistinguishable states.

Initially, let us first define a projector on an indistinguishable subspace of the system, on computational basis, as

19 $${\rm{\Pi }}=\sum _{ij}\,|ij\rangle \langle ij|,$$

with the summation running over the indistinguishable bases, i.e., those mixed during an entangling transformation. For two qubits on basis (|00〉, |01〉, |10〉, |11〉), supposing qubit pairs of same parity to become indistinguishable (say, through time reversal symmetry), one could have ${\rm{\Pi }}=\mathrm{|00}\rangle \langle \mathrm{00|}+\mathrm{|11}\rangle \langle \mathrm{11|}$ or ${\rm{\Pi }}=\mathrm{|01}\rangle \langle \mathrm{01|}+\mathrm{|10}\rangle \langle \mathrm{10|}$. In general, if one has more qubits, or multi-level units (e.g. qutrits, qudits, etc.), Π may combine more bases. On the other hand, consider an arbitrary state living in a Schmidt space, spanned by bases calculated according to the recipe described above. We can now make a projector on such space Σ given by

20 $${\rm{\Sigma }}=\sum _{k}\,|\tilde{k}\tilde{k}^{\prime} \rangle \langle \tilde{k}\tilde{k}^{\prime} |,$$

where the summation runs up to Schmidt’s rank and $\tilde{k}$ and $\tilde{k}^{\prime}$ may be equal or orthogonal depending on the system (e.g., bosonic or fermionic). One can prove that if ||Σ − Π|| < 1, the two projectors have the same rank (see Appendix 1). This is the case when the state in question lives only in the indistinguishable subspace given by the image of Π, forcing the Schmidt space to have the same rank as the number of indistinguishable bases within the projector Π (see Appendix 3). The connection between Schmidt rank and Π rank tells us that when we have indistinguishable bases in the state space (e.g., |01〉 and |10〉), we are expected to be in an entangled set-up between the bases. In other words, the indistinguishability of these bases generates entanglement between them.

One may wonder how can the linear combination of two bases account for indistinguishability, which is expected to induce linear dependence of the indistinguishable bases. When related by symmetry, the indistinguishable bases correspond to symmetric images of one another upon a certain symmetry action, and only one of them should make sense, the other becoming a correspondent in a “virtual” space, like images in a mirror. On such assumption, one should use the eigenstates of the symmetry supporting indistinguishability (in this case, Bell states) as bases to span the symmetric space, with states of the same parity related by symmetry becoming linearly dependent.

To clarify the above perspective, we divide the whole original space (without symmetry) into “symmetric” space (the relevant half sector after symmetry is imposed) and “virtual” space (the remaining half replicated by symmetry). The corresponding “virtual” bases (i.e., those corresponding to a basis by symmetry transformation) can be treated as linearly independent on the whole space combining virtual and symmetric space (see Supplementary information).

## Maximum Entanglement for Two Indistinguishable Qubits

Although Schmidt rank gives us some information about the presence of entanglement, it is not a complete measure of it. However, we may utilize the projector Π defined above to reach Tsirelson’s bound for the CHSH correlations in equation (1)^3^.

We start by expanding local operators in terms of local POVMs ${\mathscr{O}}$ as

21 $${a}_{1}^{m}=\sum _{i}\,{c}_{i}^{(m)}{{\mathscr{O}}}_{i}^{A},\,{a}_{2}^{n}=\sum _{i}\,{c}_{i}^{(n)}{{\mathscr{O}}}_{i}^{B},$$

acting on two parties *A* and *B*, and *i* identifying orthogonal POVMs spanning *a*_1_ and *a*_2_. Their direct product ${a}_{1}^{m}{a}_{2}^{n}$ gives us the total nonlocal operator representing a joint measurement on the bipartite system that will become our entanglement witnesses. We may compute the maximum expectation value for correlations based on these operators for states spanned by indistinguishable bases by taking the inner product with the projectors on the indistinguishable space Π, i.e.,

22 $$\begin{array}{rcl}\langle {a}_{1}^{m}{a}_{2}^{n}\rangle  & \le  & {\rm{Tr}}({\rm{\Pi }}{a}_{1}^{m}{a}_{2}^{n})\\  & = & {\rm{Tr}}({\rm{\Pi }}\,\sum _{ij}\,{c}_{i}^{(m)}{c}_{j}^{(n)}{{\mathscr{O}}}_{i}^{A}{{\mathscr{O}}}_{j}^{B})\\  & = & \sum _{ij}\,{c}_{i}^{(m)}{c}_{j}^{(n)}{\rm{Tr}}({\rm{\Pi }}{{\mathscr{O}}}_{i}^{A}{{\mathscr{O}}}_{j}^{B})\\  & = & \sum _{i\ne j}\,{c}_{i}^{(m)}{c}_{j}^{(n)}{\rm{Tr}}({\rm{\Pi }}{{\mathscr{O}}}_{i}^{A}{{\mathscr{O}}}_{j}^{B})\\  &  & +\,\sum _{k}\,{c}_{k}^{(m)}{c}_{k}^{(n)}\mathrm{Tr}({\rm{\Pi }}{{\mathscr{O}}}_{k}^{A}{{\mathscr{O}}}_{k}^{B})\mathrm{.}\end{array}$$

In the last equality in equation (22), the first term vanishes, and we obtain the maximum correlation when the trace in it equals unity. Since the coefficients obey normalizing conditions ${\sum }_{i}\,|{c}_{i}^{(m)}{|}^{2}={\sum }_{i}\,|{c}_{i}^{(n)}{|}^{2}=1$, one may check maximum correlations to occur when *A*_1_ and *A*_2_ have each one (mutually orthogonal) component, and *B*_1_ and *B*_2_ are spanned by orthogonal linear combinations of those, leading to the familiar $2\sqrt{2}$ Tsirelson’s bound (for detailed calculation, see Appendix 4).

While this discussion allows one to explore the role of indistinguishability within quantum mechanics, we may look for more generality beyond quantum theory. Based on sharp measurement formalism for GPTs, we shall explore the extension of indistinguishability to this broader scenario in the rest of this paper.

## Sharp Measurements

For GPTs, following Chiribella and Yuan^21^, we apply the concept of sharp measurements together with E principle to discuss *n*-body nonlocality. Sharp measurements are idealized measurements in GPT formalisms that are minimally disturbing and repeatable. In other words, a sharp measurement is assumed to be realizable on a system many times (repeatable) without influencing the result of any other compatible measurement (minimally disturbing). Also, it is possible to realize a sharp measurement by joining a measurement (not necessarily sharp) on the system and the environment together. In addition to such definition properties, two other properties arise from simple principles: (a) two sharp measurements taken together also makes a sharp measurement and (b) coarse-graining (i.e., combining various information of) a sharp measurement also gives a sharp measurement (less information, more sharpness principle). These assumptions lead to E principle (described below) as well as to the exclusive hierarchy^21^.

E principle states that if *n* events are pairwise exclusive, they must also be *n*-wise exclusive. Two events are said to be exclusive if they cannot occur at the same time, which means that they are disjunct and the summation of their probabilities must be at most unity. It has given an explanation for quantum contextuality^27^, and later was also applied to the nonlocality problem^16^, as we will discuss in more details later.

**Figure 3:**
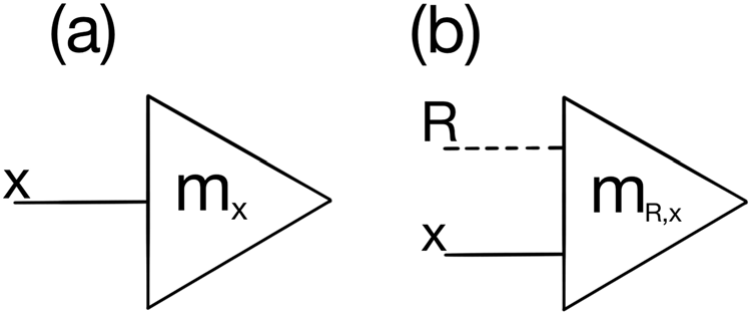
(**a**) Schematic representation of sharp measurement^21^. A sharp measurement (*m*_*x*_| on basis *x* is represented by a triangle, acting on a system by its basis *x* indicated by solid line(s). (**b**) Adjustment of the scheme in (**a**) by explicitly adding a reference *R* to gauge the measurement, represented by the dashed line.

To build a GPT footing similar to the mentioned above, we must translate sharp measurement formalism to our concept of physical reference/encoding double bit entry. A general bit (gbit) following such GPTs with sharp measurements shall be represented, modifying Chiribella and Yuan’s representation^21^, as |*r*, *b*), again having *r* to stand for its reference bit and *b* for the coding bit. Accordingly, (*m*_*R*,*x*_| shall represent effects (i.e. transformations) on such states, with *R* being the reference input and *x* denoting relevant bases (Fig. 3). The coding information in *b* is defined regarding reference *r* as is the effect gauged by some *R*. It differs from Chiribella and Yuan^21^ by explicitly adding the reference input that is tacitly assumed in the concept of sharp measurements. For a sharp measurement on multiple parties, either one or multiple references may be present in principle, though not more than one per retrieved information bit, for consistency.

Above mentioned assumptions on joint sharp measurements (a) and coarse-graining (b) can be readily generalized for our approach. Joining measurements (*m*_*R*,*x*_| and (*n*_*R*′,*y*_| may be represented by writing $({m}_{R,x}|\otimes ({n}_{R^{\prime} ,y}|=({m^{\prime} }_{RR^{\prime} ,xy}|$, though the precise method for computing it is irrelevant. Coarse graining may be thought of in two manners: (i) coding bits with the same physical reference may be coarse-grained or (ii) a pair of coding bits and their references may be coarse-grained together in any situation. We will use it to revisit E principle application to *n*-body non-locality.

## Information Indistinguishability and E Principle

Cabello has used E principle to show that quantum bounds of *n*-body nonlocality derived by Collins *et al*.^7^ and Seevinck and Svetlichny^8^ can be explained by such probabilistic principle. The inequality in question,

23 $$|\langle {S}_{n}\rangle |{\le }_{{\rm{QT}}}{2}^{n-1}\sqrt{2}{\le }_{{\rm{NS}}}{2}^{n},$$

limits the correlation bound for quantum theory, identified by the subscript “QT,” and the limits for a no-signaling correlation box which is the same as the mathematical limit for the equation, identified with subscript “NS”.

Owing to *S*_*n*_ recursive definition, one can immediately understand its bound if one knows the bounds for |〈*S*_2_〉|. However, one can also try to obtain the $\sqrt{2}$ factor for the *n*-body correlation bounds directly from an *n*-body system. This is what Cabello does^16^, for instance. Information indistinguishability also offers insights in this perspective. Consider, for instance, the bounds for $|\langle {S}_{n}^{2}\rangle |{\le }_{{\rm{QT}}}{2}^{2n-1}{\le }_{{\rm{NS}}}{2}^{2n}$. Mathematically, it corresponds merely to the square of equation 23. Physically, one can conceive such scenario by taking two identical copies of the same system, say *A* and *A*′. In this case, we can show the quantum bound under indistinguishability considerations.

For two identical copies of a system, we can calculate the whole system *A* ⊗ *A*′ maximum correlation as $|\langle {S}_{n}^{2}\rangle |$, for only correlations in at most an *n*-body subsystem are supposed to exist. In terms of the presented indistinguishability, one can think of *A* and *A*′ as indistinguishable systems, with *A* as the physical reference and *A*′ the encoding space supposed indistinguishable of *A* and with parity constrained by it. This essentially assumes coarse-graining of *A* and *A*′ to a single gbit each, and general “parity” either odd or even. In this case, we have 2^*n*^ “contexts” for the basis chosen for *A*, and more 2^*n*^ for *A*′, but only half of those are allowed as non-vanishing correlation term for each context in *A*, according to global parity. Because each term assumes maximum correlation of 1, the maximum correlation becomes $|\langle {S}_{n}^{2}\rangle |\le {2}^{n}\times {2}^{n}/2={2}^{2n-1}$. Alternatively, once gbits are rendered indistinguishable in the entanglement process, we can pick one of them to serve us as a global reference, itself defining two measurement outcomes. The remaining 2*n* − 1 gbits (or bases of measurement) must have the same overall parity as defined by the reference bit, leading to 2 × 2^2*n*−1^/2 non-vanishing correlation terms. Once again, the maximum algebraic correlation allowed for such case becomes $|\langle {S}_{n}^{2}\rangle |\le {2}^{2n-1}$ or $|\langle {S}_{n}\rangle |\le {2}^{n-1}\sqrt{2}$, retrieving once more Tsirelson’s bound.

For the application of E principle, an extra assumption must be made. Besides the assumptions that define sharp measurements, one assumes the existence of a sharp measurement *A*_*ij*_ s.t. *A*_00_ and *A*_11_ are compatible and *A*_01_ and *A*_10_ are also compatible. *A*_*ij*_ is composed by sharp measurements $({x}_{1}^{0}|$ and $({x}_{1}^{1}|$ on system *A* and $({x}_{1^{\prime} }^{\,0}|$ and $({x}_{1^{\prime} }^{\,1}|$ on system *A*′, yielding 0 if measurements $({x}_{1}^{i}|$ and $({x}_{1^{\prime} }^{\,j}|$ return the same result and 1 otherwise. This assumption is used to derive quantum bounds to equation (23) from two copies of a system, *A* and *A*′. This assumption can be justified by information indistinguishability.

Let a system *A* and a copy *A*′ of it be prepared and represented by |*b*_1_, *b*_2_, …) and $|{b^{\prime} }_{1},{b^{\prime} }_{2},\ldots )$ respectively, where the prime becomes the reference bit for the system. Systems *A* and *A*′ may be located far apart, which is the information indicated by the prime, and are therefore distinguishable in principle. The composite system *AA*′ can then be represented by $|{b}_{1},{b^{\prime} }_{1};{b}_{2},{b^{\prime} }_{2};\ldots )$. By definition, *A*_*ij*_ is a measurement that does not differentiate gbits *b*_1_ and ${b^{\prime} }_{1}$. We may therefore drop the background information signalized by the prime bit, rewriting the state as $|{b}_{1},{\tilde{b}}_{1};{b}_{2},{\tilde{b}}_{2};\ldots )$, where bits *b*_*i*_ and ${\tilde{b}}_{i}$ become mutual reference and coding information. This implies equivalence between states |00) and |11) and states |01) and |10). This state equivalence assures that there are at least two compatible measurements to each indistinguishable scenario, namely *A*_00_/*A*_11_ and *A*_01_/*A*_10_. This is enough to assure tight quantum bounds in equation (23), with a brief derivation given in Appendix 5. For a detailed derivation, see Cabello^16^.

## Conclusion and Discussion

Information indistinguishability provides an interpretation that extends the indistinguishability of (identical) particles to information units like qubits or an equivalent binary state in GPTs we refer to as gbits. In quantum theory, it can be understood as a dissociation of encoding internal degrees of freedom of a qubit, rendered indistinguishable if taken alone, and their real physical properties like charge, mass, position and so on that allows qubits to be distinct. By explicitly separating such information into a physical background reference and an internal coding bit, we may ignore such reference when their internal degrees of freedom are coupled and assume their new reference to become one another in a pair of qubits.

In a more general perspective, we can analyze general bits on GPTs by using sharp measurements and E principle implied by it. We observe that information indistinguishability sustains the existence of non-local sharp measurements that may be used to complete inequalities to derive tight quantum bounds for nonlocality. This supports the consideration of information indistinguishability as a fundamental physical principle generator and restrictor of entanglement. Indeed, a Popescu-Rohrlich-like correlation box that extrapolates quantum nonlocality violates information indistinguishability. For example, consider two gbits and two measurements each, where three measurement pairings give perfect correlation and one pairing no correlation at all, hence 〈*S*_2_〉 = 3. By pinning one axes combination to lack correlation, the particles are identified along these axes, for if we assume that gbits become correlated when they are indistinguishable, the non-correlated axis will allow to track down the correlation process, identifying each party during coupling, contradicting our hypothesis. Such lack of transitivity between perfect correlations (viz. measurements ${a}_{1}^{0}$ and ${a}_{1}^{1}$ perfectly correlate with ${a}_{2}^{0}$, ${a}_{2}^{1}$ perfectly correlate with ${a}_{1}^{0}$, but such correlation does not extend to ${a}_{1}^{1}$ and ${a}_{2}^{1}$) is unrealistic and incompatible with information indistinguishability. Similarly, supersymmetric qubits can use odd (anticommuting) dimensions to identify each qubit in an entangled state, attributing different amplitudes for superpositions between even and odd states^28^ and breaking indistinguishability. These incompatibilities suggest the consideration of information indistinguishability as an indicator for realistic theories.

This approach suggests information indistinguishability as means for nonlocal realistic entanglement, offering a relatively simple understanding for the limitation of realistic nonlocality in quantum theory.

## A Lemma and Proof

### Lemma.

*Given two projectors P and Q* obeying ||*P* − *Q*|| < 1, ${\rm{rank}}\,P={\rm{rank}}\,Q$.

This lemma and proof is provided by Dym^29^. If *P* and *Q* are two projectors and ||*P* − *Q*|| < 1, clearly *I*_*n*_ − (*P* − *Q*) is full rank and invertible. Hence:

24 $$\begin{array}{rcl}{\rm{rank}}\,P & = & {\rm{rank}}\,P({I}_{n}-(P-Q))={\rm{rank}}\,PQ\\  & \le  & {\rm{rank}}\,Q\mathrm{.}\end{array}$$

The second equality comes from projectors’ idempotency (*P*^2^ = *P*). Since the same argument can be made exchanging *P* and *Q*, they must have the same rank. QED.

## B Schmidt Decomposition

Given a bipartite quantum system, it can, in general, be represented as

25 $$|{\rm{\Psi }}\rangle =\sum _{ij}\,{c}_{ij}|i\rangle |j\rangle \mathrm{.}$$

By performing a singular value decomposition (SVD) of the coefficient matrix, one obtains

26 $$\begin{array}{rcl}|{\rm{\Psi }}\rangle  & = & \sum _{ijk}\,{v}_{ik}\sqrt{{\lambda }_{k}}\,{u}_{kj}|i\rangle |j\rangle \\  & = & \sum _{k}\,\sqrt{{\lambda }_{k}}|{\lambda }_{k}^{A}\rangle |{\lambda }_{k}^{B}\rangle ,\end{array}$$

where $\sqrt{{\lambda }_{k}}$ are the singular values, and the correlated kets in the second line are obtained by applying *v*_*ik*_ (*u*_*kj*_) to |*i*〉 (|*j*〉). These are called Schmidt bases, and the rank of the matrix ${\rm{\Lambda }}=(\sqrt{{\lambda }_{k}})$, i.e., the number of singular values, is called Schmidt rank. A Schmidt rank equal to 1 implies that the quantum state is separable into the product of two independent states, and is therefore unentangled by definition. For this reason, Schmidt rank serves as a measure of entanglement. For more, see, for instance, Nielsen and Chuang^25^.

The SVD performed above implicitly assumes identification of the kets in the case by ordering. For identical particles and an extension for indistinguishable states, a recipe is given by Sciara *et al*.^18^. First, a partial trace is done on the density matrix *ρ* down to a single particle reduced density matrix *ρ*^(1)^. For this, a symmetric inner product is performed, following equation (5). Then, by diagonalizing *ρ*^(1)^ the obtained eigenstates |*i*〉 generate our Schmidt basis $|\tilde{i},\tilde{i}^{\prime} \rangle$, with the relevant singular value amounting to their contribution being the square root of the eigenvalues. This approach gives a slightly different result than expected by straightforward SVD and becomes more consistent with particle statistics.

## C Proof of Schmidt’s Rank Raise

For the Schmidt space projector ${\rm{\Sigma }}={\sum }_{k}\,|{\lambda }_{k}\rangle \langle {\lambda }_{k}|$ defined in the main text, one can revert it to the same basis as an *r*-rank (*r*>1) projector Π by reverting Schmidt decomposition. If an SVD approach is used, one has that:

27 $$\begin{array}{rcl}{\rm{\Sigma }} & = & \sum _{k}\,|{\lambda }_{k}\rangle \langle {\lambda }_{k}|\\  & = & \sum _{ijk}\,{v}_{ik}{u}_{jk}|ij\rangle \langle ij|{v}_{ik}^{\ast }{u}_{jk}^{\ast }\\  & = & \sum _{ijk}\,|{v}_{ik}{|}^{2}|{u}_{jk}{|}^{2}|ij\rangle \langle ij\mathrm{|.}\end{array}$$

If we suppose that only one Schmidt basis exists, the summation over *k* is trivial, and |*v*_*i*_| = |*u*_*j*_| = 1 in order to have a norm 1 projector. Then, we obtain

28 $${\rm{\Sigma }}-{\rm{\Pi }}=\sum _{ij\notin {\rm{\Pi }}}\,|ij\rangle \langle ij\mathrm{|.}$$

If Σ contains bases not in Π, we still have a norm 1 operator, and a non-entangled state is possible, as assumed. However, if only the bases in Π are present, this should be a norm 0 operator, and according to lemma 1, should bear the same rank as Π. Indeed, if one does not ignore the summation over *k* and assumes |*v*_*ik*_|, |*u*_*jk*_| < 1, a finite component of this difference remains, but still one has ||Σ − Π|| < 1, reassuring that *k* = *r* > 1. Hence, for a state within Π’s range, Schmidt rank is greater than 1.

Note that though SVD assumes identification of the states in question, in this case, indistinguishability is introduced ad-hoc with projector Π, making the above demonstration still valid. One may nonetheless use the transformations discussed for the case of identical bases. In this case, Schmidt decomposition of each of the indistinguishable bases gives the same Schmidt bases and therefore the same projector Σ. When returning from Schmidt bases to computational bases, the underlying ambiguity forces one to take a linear combination much in the same fashion as the one written above, leading to essentially the same calculation.

## D Two-qubit Maximum Correlation

Equation (22) in the main text takes for its maximum value

29 $$\sum _{k}\,{c}_{k}^{(m)}{c}_{k}^{(n)},$$

when a state lives in the range of the operator Π. One can simply analyze the possible expansions of operators ${a}_{1}^{m}$ and ${a}_{2}^{n}$ according to equation (21). The simplest case occurs for only two *a*_1_s and *a*_2_s, which must be represented by the same local operators ${{\mathscr{O}}}_{1}^{i}$ and ${{\mathscr{O}}}_{2}^{i}$ (*i* indicating *A* or *B*) to bear any correlation. In this case, each *c*_*k*_ = 1 and 〈*S*_2_〉 = 2. We may also consider the case of one of the operator alone having two components, but this will not change much since only one of the components will be non-orthogonal to the other operators and generate correlations.

We may then consider the case of two operators with two components. First, suppose the case of ${a}_{1}^{0}={{\mathscr{O}}}_{1}$, ${a}_{2}^{0}={{\mathscr{O}}}_{1}$, ${a}_{1}^{1}={c}_{i}^{A}{{\mathscr{O}}}_{1}+{c}_{2}^{A}{{\mathscr{O}}}_{2}$, and ${a}_{2}^{1}={c}_{i}^{B}{{\mathscr{O}}}_{1}+{c}_{2}^{B}{{\mathscr{O}}}_{2}$. Their maximum correlations become

30 $$\begin{array}{ll}\langle {a}_{1}^{0}{a}_{2}^{0}\rangle =\pm \,1, & \langle {a}_{1}^{0}{a}_{2}^{1}\rangle =\pm {c}_{1}^{B}\\ \langle {a}_{1}^{0}{a}_{2}^{0}\rangle =\pm \,{c}_{1}^{A}, & \langle {a}_{1}^{1}{a}_{2}^{1}\rangle =\pm \,{c}_{1}^{A}{c}_{1}^{B}\pm {c}_{2}^{A}{c}_{2}^{B},\end{array}$$

which leads to global correlations of the form

31 $$\langle {S}_{2}\rangle =1+{c}_{1}^{A}+{c}_{1}^{B}-{c}_{1}^{A}{c}_{1}^{B}\pm {c}_{2}^{A}{c}_{2}^{B}\mathrm{.}$$

**Figure 4:**
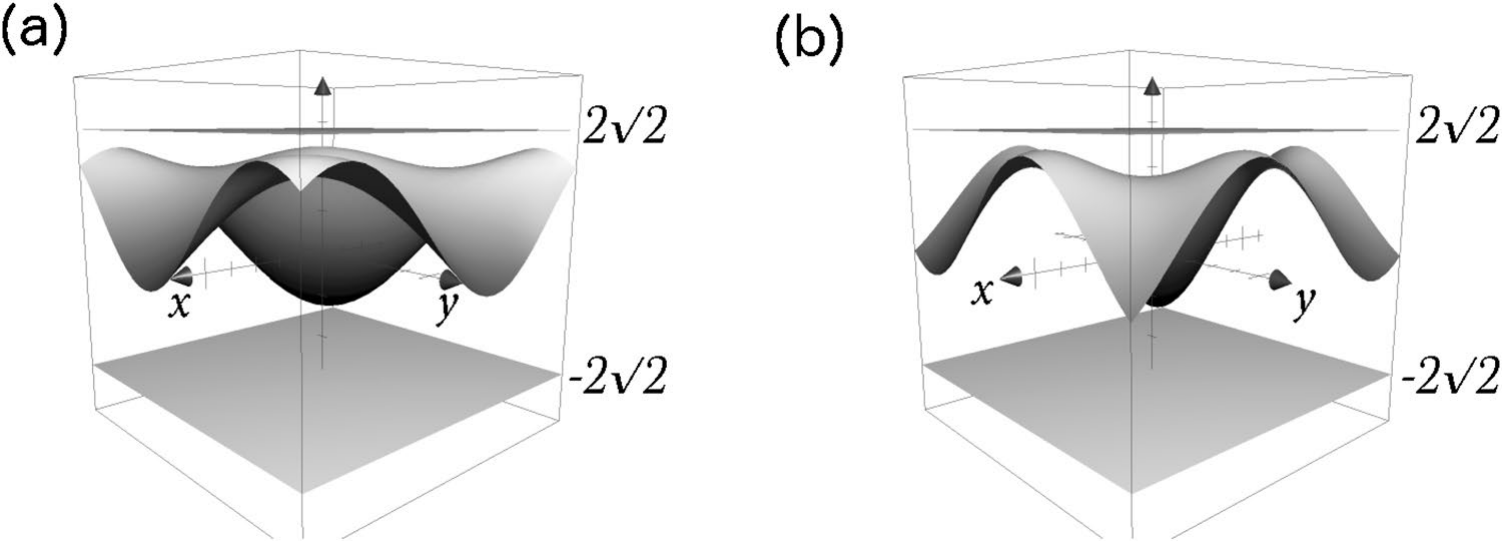
Correlation function in equation (31) taken (**a**) +, (**b**) −. Given the normalization condition, ${c}_{1}^{A(B)}$ is rewritten as sin *x*(*y*) and ${c}_{2}^{A(B)}$ as cos *x*(*y*). The upper and lower plan indicate $z=\pm \,2\sqrt{2}$, and axes range from −*π* to *π*.

Since normalization conditions require that $|{c}_{1}^{A}{|}^{2}+|{c}_{2}^{A}{|}^{2}=1$ and $|{c}_{1}^{B}{|}^{2}+|{c}_{2}^{B}{|}^{2}=1$, one can place the substitutions ${c}_{1}^{A}=\,\sin \,x,\,{c}_{2}^{A}=\,\cos \,x,\,{c}_{1}^{B}=\,\sin \,y,\,{c}_{2}^{B}=\,\cos \,y$ and calculate the highest correlation achieved to be $1+\sqrt{2}$, which can also be verified numerically by plotting 〈*S*_2_〉 as in Fig. 4.

Another two-component possibility lies on the case $${a}_{1}^{0}={{\mathscr{O}}}_{1},\,{a}_{1}^{1}={{\mathscr{O}}}_{2},\,{a}_{2}^{0}={c}_{1}^{\mathrm{(1)}}{{\mathscr{O}}}_{1}+{c}_{2}^{\mathrm{(1)}}{{\mathscr{O}}}_{2},\,{a}_{2}^{1}=$$$${c}_{1}^{\mathrm{(2)}}{{\mathscr{O}}}_{1}+{c}_{2}^{\mathrm{(2)}}{{\mathscr{O}}}_{2}$$. The correlations for such operators can be calculated as

32 $$\begin{array}{ll}\langle {a}_{1}^{0}{a}_{2}^{0}\rangle =\pm \,{c}_{1}^{\mathrm{(1)}}, & \langle {a}_{1}^{0}{a}_{2}^{1}\rangle =\pm \,{c}_{1}^{\mathrm{(2)}}\\ \langle {a}_{1}^{1}{a}_{2}^{0}\rangle =\pm \,{c}_{2}^{\mathrm{(1)}}, & \langle {a}_{1}^{1}{a}_{2}^{1}\rangle =\pm \,{c}_{2}^{\mathrm{(2)}},\end{array}$$

and

33 $$\langle {S}_{2}\rangle ={c}_{1}^{\mathrm{(1)}}+{c}_{1}^{\mathrm{(2)}}+{c}_{2}^{\mathrm{(1)}}-{c}_{2}^{\mathrm{(2)}}\mathrm{.}$$

By using the same substitution as in the previous case, it is straightforward to show that $|\langle {S}_{2}\rangle |\le 2\sqrt{2}$. If more components are assumed, the contribution of each component to the total correlation decreases, and the maximum value for 〈*S*_2_〉 decreases together, unless every component takes part on the total correlation 〈*S*_2_〉, leaving the maximum value of the known Tsirelson bound. Consider, for instance, the case of ${a}_{1}^{0}={c}_{1}{{\mathscr{O}}}_{1}+{c}_{2}{{\mathscr{O}}}_{2},\,{a}_{1}^{1}={{\mathscr{O}}}_{3},$ ${a}_{2}^{0}={d}_{1}{{\mathscr{O}}}_{1}+{d}_{2}{{\mathscr{O}}}_{2}+{d}_{3}{{\mathscr{O}}}_{3},\,{a}_{2}^{1}={d^{\prime} }_{1}{{\mathscr{O}}}_{1}+{d^{\prime} }_{2}{{\mathscr{O}}}_{2}+{d^{\prime} }_{3}{{\mathscr{O}}}_{3}$. In this case, we can calculate 〈*S*_2_〉 to be

34 $$\langle {S}_{2}\rangle ={c}_{1}{d}_{1}+{c}_{2}{d}_{2}+{c}_{1}{d^{\prime} }_{1}+{c}_{2}{d^{\prime} }_{2}+{d}_{3}-{d^{\prime} }_{3}\mathrm{.}$$

We may assume, to maximize 〈*S*_2_〉, that terms sharing the same sign related to the same operator, e.g. *c*_1_*d*_1_ and *c*_1_${d^{\prime} }_{1}$, to hold the same value and double their correlation. Equally, related terms of opposite sign (*d*_3_ and −${d^{\prime} }_{3}$), can also double themselves by assuming opposite value to each coefficient (*d*_3_ = −${d^{\prime} }_{3}$). Therefore, one can expect that

35 $$|\langle {S}_{2}\rangle |\le \mathrm{2(}{c}_{1}{d}_{1}+{c}_{2}{d}_{2}+{d}_{3}\mathrm{).}$$

**Figure 5:**
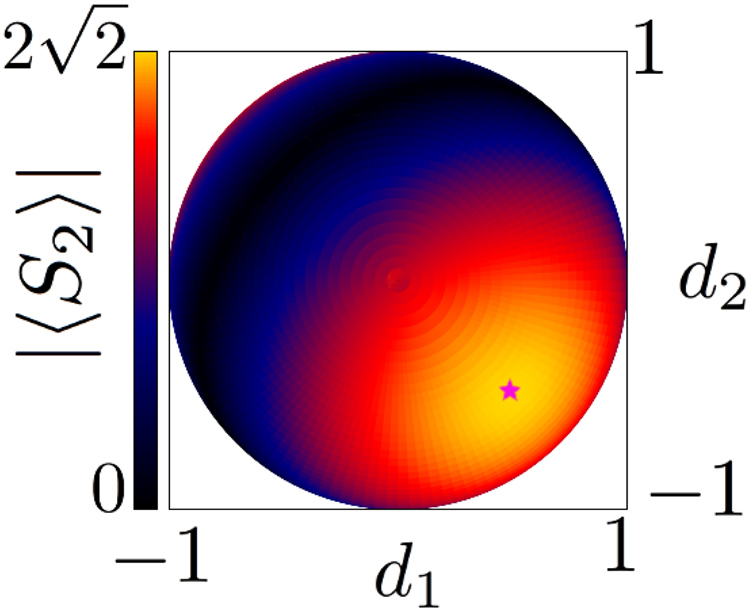
Top view of a sphere defined by parameters ${d}_{1}^{2}+{d}_{2}^{2}+{d}_{3}^{2}=1$ in equation (35) with color plot of |〈*S*_2_〉|. Parameters for qubit *A* measurement are fixed at ${c}_{1}={c}_{2}=1/\sqrt{2}$. Color indicates the value of |〈*S*_2_〉| on a point on the sphere, reaching Tsirelson’s bound at its maximum, marked by the pink star on the figure (gold color plot).

We can maximize this correlation by assuming an even distribution of expectation values between operators ${\mathscr{O}}$ so that no information is lost in terms that cannot be measured or that may cancel one another. Explicitly, in this case, this would require ${c}_{1}={c}_{2}=1/\sqrt{2}$, and *d*_1_ = ${d^{\prime} }_{1}$ = *d*_2_ = ${d^{\prime} }_{2}$ = 1/2 and ${d}_{3}=-\,{d^{\prime} }_{3}=1/\sqrt{2}$. A plot of this correlation is given in Fig. 5. In general, Tsirelson’s bound is reached at the point where each *S*^*m*^ × *S*^*n*^ manifold (i.e., *m*-sphere × *n*-sphere) spanned by (*m* + 1) × (*n* + 1) components in a joint measurement of *A* and *B* (for example, for the ${a}_{1}^{0}{a}_{2}^{0}$ term, the *S*^1^ × *S*^2^ manifold spanned by *c*_1_, *c*_2_ from ${a}_{1}^{0}$ and *d*_1_, *d*_2_, *d*_3_ from ${a}_{2}^{0}$) has the expectation value of the joint measurement evenly distributed between all components. For example, Fig. 5 fixes a point on the *S*^1^ manifold where contribution of parameters *c*_1_ and *c*_2_ are maxed out, and shows the distribution of possible correlations on the *S*^2^ manifold spanned by parameters *d*_1_, *d*_2_, *d*_3_, so that each joint manifold *S*^1^ × *S*^2^ reaches a maximum at $\mathrm{(1/2},\mathrm{1/2},1/\sqrt{2})$ and $(\,-\,\mathrm{1/2},-\,\mathrm{1/2},-\,1/\sqrt{2})$ on (*d*_1_, *d*_2_, *d*_3_) or (${d^{\prime} }_{1}$, ${d^{\prime} }_{2}$, −${d^{\prime} }_{3}$) basis.

It can be verified that the maximum correlations happen when no POVM ${\mathscr{O}}$ spanning measurements on parties *A* and *B* give zero expectation value (for this would limit the maximum contribution of remaining POVMs), nor they cancel other POVMs’ expectation value. Hence, for *n* orthogonal POVMs, we may take operators acting on *B* to have a general form ${a}_{2}^{0}={\sum }_{i=1}^{n}\,{d}_{i}{{\mathscr{O}}}_{i}^{B}$ and ${a}_{2}^{1}={\sum }_{i=1}^{n}\,{d^{\prime} }_{i}{{\mathscr{O}}}_{i}^{B}$, and adjust measurements on *A* such that all components ${{\mathscr{O}}}_{i}$ are distributed among ${a}_{1}^{0}$ and ${a}_{1}^{1}$, and no ${{\mathscr{O}}}_{i}$ appears in both of them, i.e. ${a}_{1}^{0}={\sum }_{i=1}^{k < n}\,{c}_{i}{{\mathscr{O}}}_{i}^{A}$ and ${a}_{1}^{1}={\sum }_{i > k}^{n}\,{c^{\prime} }_{i}{{\mathscr{O}}}_{i}^{A}$. Therefore, with 4 ${{\mathscr{O}}}_{i}$s, one can take ${a}_{1}^{0}={c}_{1}{{\mathscr{O}}}_{1}+{c}_{2}{{\mathscr{O}}}_{2},\,{a}_{1}^{1}={c^{\prime} }_{3}{{\mathscr{O}}}_{3}+{c^{\prime} }_{4}{{\mathscr{O}}}_{4},$ ${a}_{2}^{0}={d}_{1}{{\mathscr{O}}}_{1}+{d}_{2}{{\mathscr{O}}}_{2}+{d}_{3}{{\mathscr{O}}}_{3}+{d}_{4}{{\mathscr{O}}}_{4},$ ${a}_{2}^{1}={d^{\prime} }_{1}{{\mathscr{O}}}_{1}+{d^{\prime} }_{2}{{\mathscr{O}}}_{2}+{d^{\prime} }_{3}{{\mathscr{O}}}_{3}+{d^{\prime} }_{4}{{\mathscr{O}}}_{4},$ which will give |〈*S*_2_〉|/2 ≤ *c*_1_*d*_1_ + *c*_2_*d*_2_ + ${c^{\prime} }_{3}$*d*_3_ + ${c^{\prime} }_{4}$*d*_4_. Once again, the maximum value is bounded by an even distribution on the *S*_1_ manifolds spanned by *c*s, i.e., ${c}_{1}={c}_{2}={c^{\prime} }_{3}={c^{\prime} }_{4}=1/\sqrt{2}$, and similar for *d*’s *S*^3^ manifold, with *d*_1_ = *d*_2_ = *d*_3_ = *d*_4_ = ${d^{\prime} }_{1}$ = ${d^{\prime} }_{2}$ = −${d^{\prime} }_{3}$ = −${d^{\prime} }_{4}$ = 1/2. Using the same notation for 5 components, one would have |〈*S*_2_〉|/2 ≤ *c*_1_*d*_1_ + *c*_2_*d*_2_ + *c*_3_*d*_3_ + ${c^{\prime} }_{4}$*d*_4_ + ${c^{\prime} }_{5}$*d*_5_, which reaches its maximum value at *c*_1_ = *c*_2_ = 1/2 and ${c}_{3}=1/\sqrt{2}$, ${c^{\prime} }_{4}={c^{\prime} }_{5}=1/\sqrt{2}$, and ${d}_{1}={d}_{2}={d^{\prime} }_{1}={d^{\prime} }_{2}=1/2\sqrt{2}$ and *d*_3_ = *d*_4_ = *d*_5_ = ${d^{\prime} }_{3}$ = −${d^{\prime} }_{4}$ = −${d^{\prime} }_{5}$ = 1/2. Note that while we implicitly assume real coefficients, the complex case is analogous.

## E Nonlocality Bound of ***n***-body Correlation

By rewriting *S*_*n*_ as a function of outcomes equal to 0/1 instead of −1/1, one obtains that

36 $$|\langle {\tilde{S}}_{n}\rangle |{\le }_{{\rm{QT}}}{\mathrm{(2}}^{n-2}\times 2+\sqrt{2}),$$

37 $${\tilde{S}}_{n}=\frac{{S}_{n}}{2}+{2}^{n-1}$$

38 $$\begin{array}{rcl}\langle {\tilde{S}}_{n}\rangle  & = & \sum _{\begin{array}{c}({x}_{1},{x}_{2},{x}_{3})\ne ({x}_{1},{x}_{1},{x}_{1})\\ {b}_{1}\oplus \cdots \oplus {b}_{n}=0\end{array}}p({b}_{1},\ldots ,{b}_{n}|{x}_{1},\ldots ,{x}_{n})\\  &  & +\sum _{\begin{array}{c}({x}_{1},{x}_{2},{x}_{3})=({x}_{1},{x}_{1},{x}_{1})\\ {b}_{1}\oplus \cdots \oplus {b}_{n}=1\end{array}}p({b}_{1},\ldots ,{b}_{n}|{x}_{1},\ldots ,{x}_{n}),\end{array}$$

where *b*_*i*_ ∈ {0, 1} are the outputs of the measurement on the *x*_*i*_ basis (*b* and *x* are in the inverse order of the main text, whence we add *p* for standard probability notation). By taking two copies of a system, *A* and *A*′, a global events’ probability can be written as

39 $$\begin{array}{l}p(b\mathrm{1,}\ldots ,{b}_{n},{b^{\prime} }_{1},\ldots ,{b^{\prime} }_{n}|{x}_{1},\ldots ,{x}_{n},{x^{\prime} }_{1},\ldots ,{x^{\prime} }_{n})\\ \begin{array}{rcl} & = & p({b}_{1},\ldots ,{b}_{n}|{x}_{1},\ldots ,{x}_{n})\times p({b^{\prime} }_{1},\ldots ,{b^{\prime} }_{n}|{x^{\prime} }_{1},\ldots ,{x^{\prime} }_{n}\mathrm{).}\end{array}\end{array}$$

Summation of events in ${\tilde{S}}_{n}$ within both copies gives ${\tilde{S}}_{n}^{2}$. If both copies are not in ${\tilde{S}}_{n}$, their summation adds up to ${{\mathrm{(2}}^{n}-{\tilde{S}}_{n})}^{2}$, where the 2^*n*^ comes from the 2^*n*^ different (*x*_1_, …, *x*_*n*_).

In total, there are 4^*n*^ × 4^*n*^/2 events divided into 4^*n*^ disjoint sets, each containing 4^*n*^/2 pairwise exclusive events. One may add to each set a new event $(p,q|{A}_{ij},{A}_{\bar{i}\bar{j}})$, with compatible ${A}_{ij},\,{A}_{\bar{i}\bar{j}}$ (i.e., *A*_00_, *A*_11_ or *A*_01_, *A*_10_) while keeping exclusivity. From E principle, the sum of a set of pairwise exclusive events cannot exceed 1, what translates into an inequality we may call “E inequality”. Since there are 4^*n*^ such set, summing them gives

40 $$\langle {\tilde{S}}_{n}^{2}\rangle +{{\mathrm{(2}}^{n}-\langle {\tilde{S}}_{n}\rangle )}^{2}+{4}^{n-1}\le {4}^{n}\mathrm{.}$$

The 4^*n*−1^ term comes from the addition of $(p,q|{A}_{ij},{A}_{\bar{i}\bar{j}})$ events that add to unity for a fixed *ij*, existing 4^*n*−1^ of them. From this inequality, one obtains

41 $$|\langle {\tilde{S}}_{n}\rangle |\le {2}^{n-2}\times (2+\sqrt{2})\mathrm{.}$$

For a detailed derivation, see Cabello^16^.

## Electronic supplementary material

Supplement
