# Supplementary material for: Indistinguishability as nonlocality constraint
Source: Sci Rep. 2018 Apr 17;8:6091. doi: 10.1038/s41598-018-24489-7 (PMC5904208; doi:10.1038/s41598-018-24489-7)

Indistinguishability as nonlocality constraint (Supplementary material)

Cássio S. Amorim

Consider two orthogonal vectors, and a mirror (red dotted line) between them, making them mutual mirror images

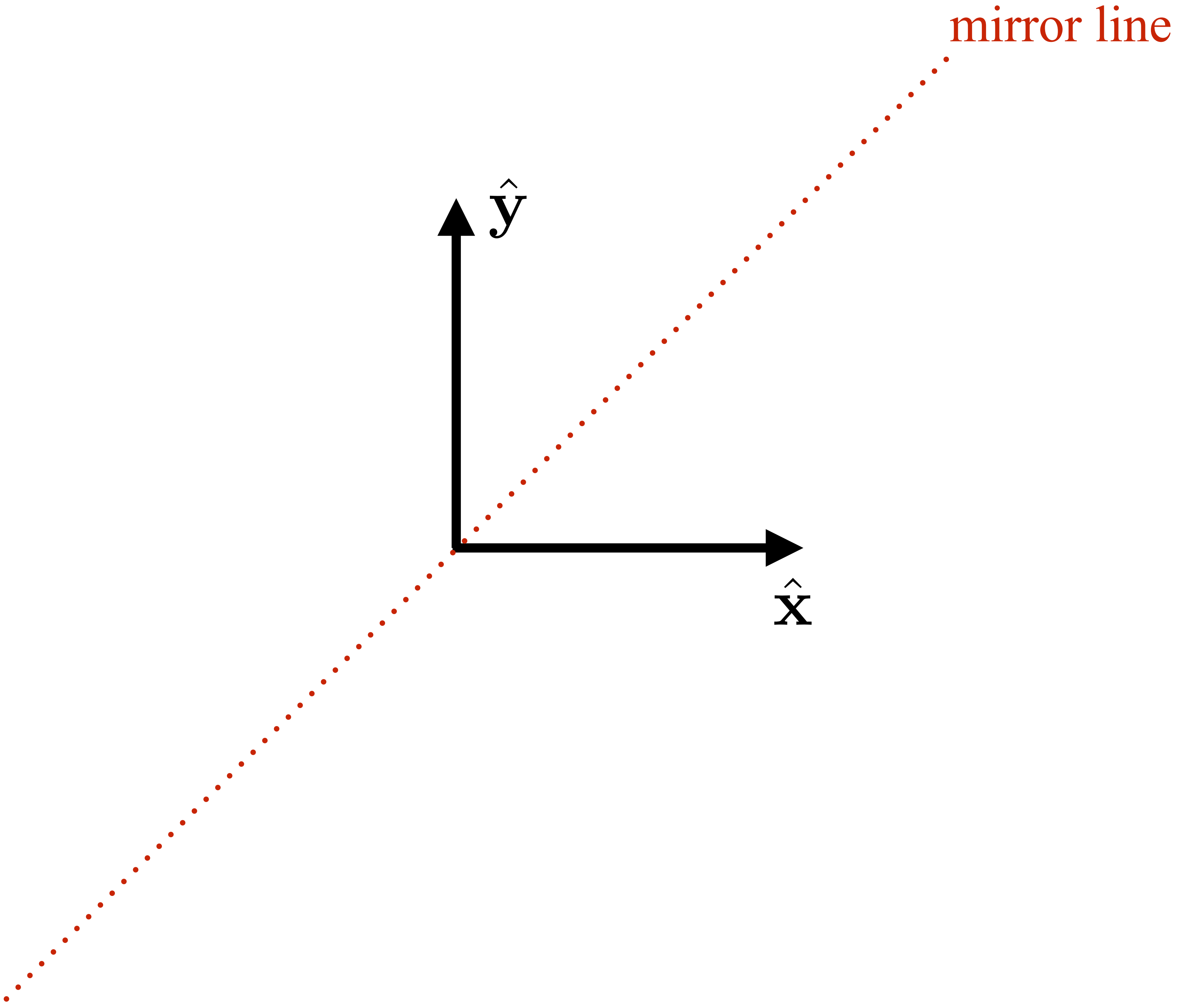

One may consider only half-plane on one side of the mirror as "real" and the other half as virtual.
By folding one half-place onto another, we may treat $\hat{\mathbf{x}} = \hat{\mathbf{y}} = \hat{\mathbf{t}} + \hat{\mathbf{n}}$

$\hat{\mathbf{t}}, \hat{\mathbf{n}}$ are eigenvectors of the mirror line spanning the half-plane

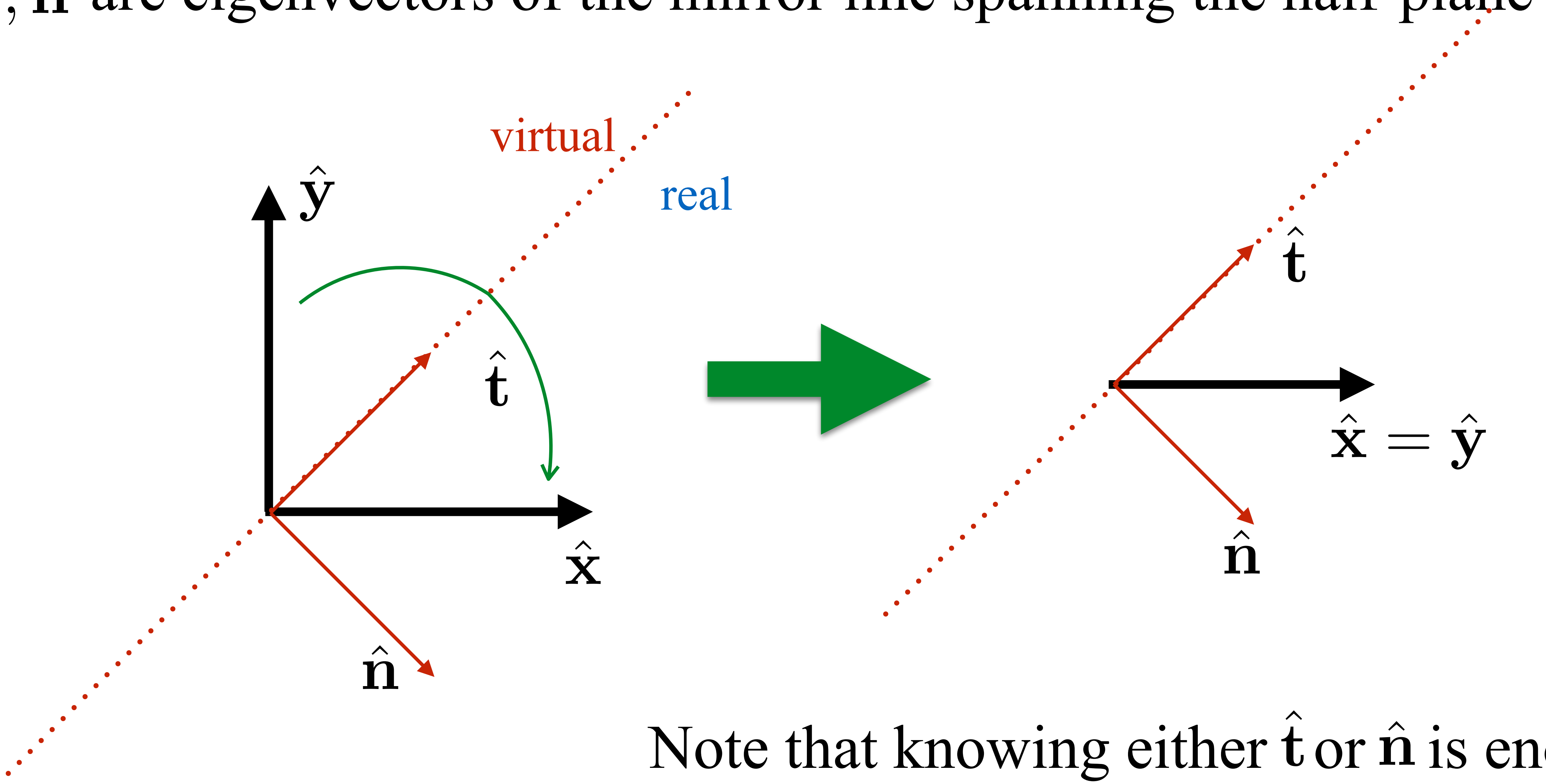

Note that knowing either $\hat{\mathbf{t}}$ or $\hat{\mathbf{n}}$ is enough for describing the symmetric space

Alternatively, one can unfold the half-planes and write both real and virtual spaces and their vectors as linearly independent. In this case:

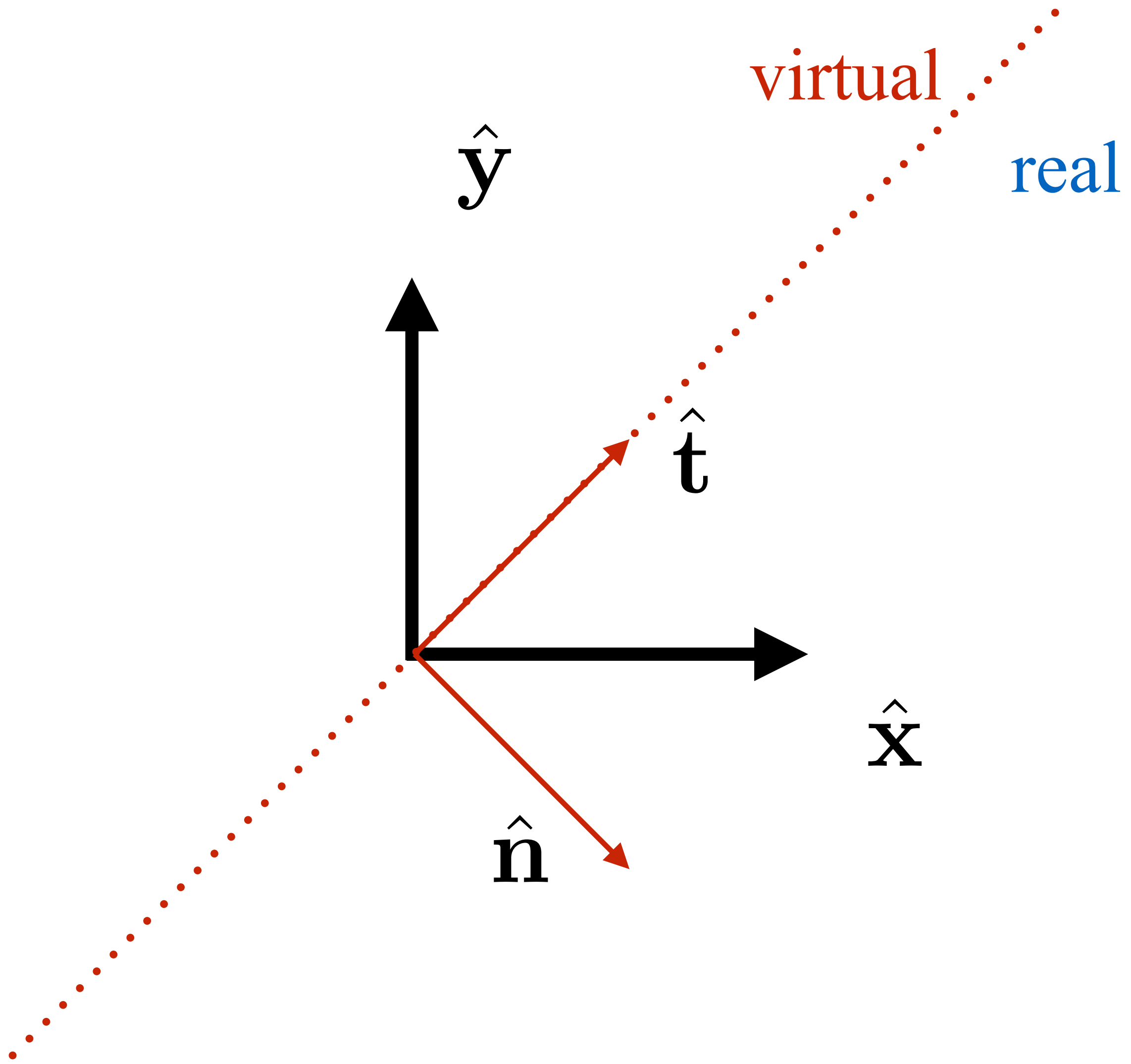

$$\hat{\mathbf{x}} = \hat{\mathbf{t}} + \hat{\mathbf{n}}$$
$$\hat{\mathbf{y}} = \hat{\mathbf{t}} - \hat{\mathbf{n}}$$

Sign indicates virtual vector

One can reread the mirror as a generic symmetry mapping orthogonal state vectors, for instance:

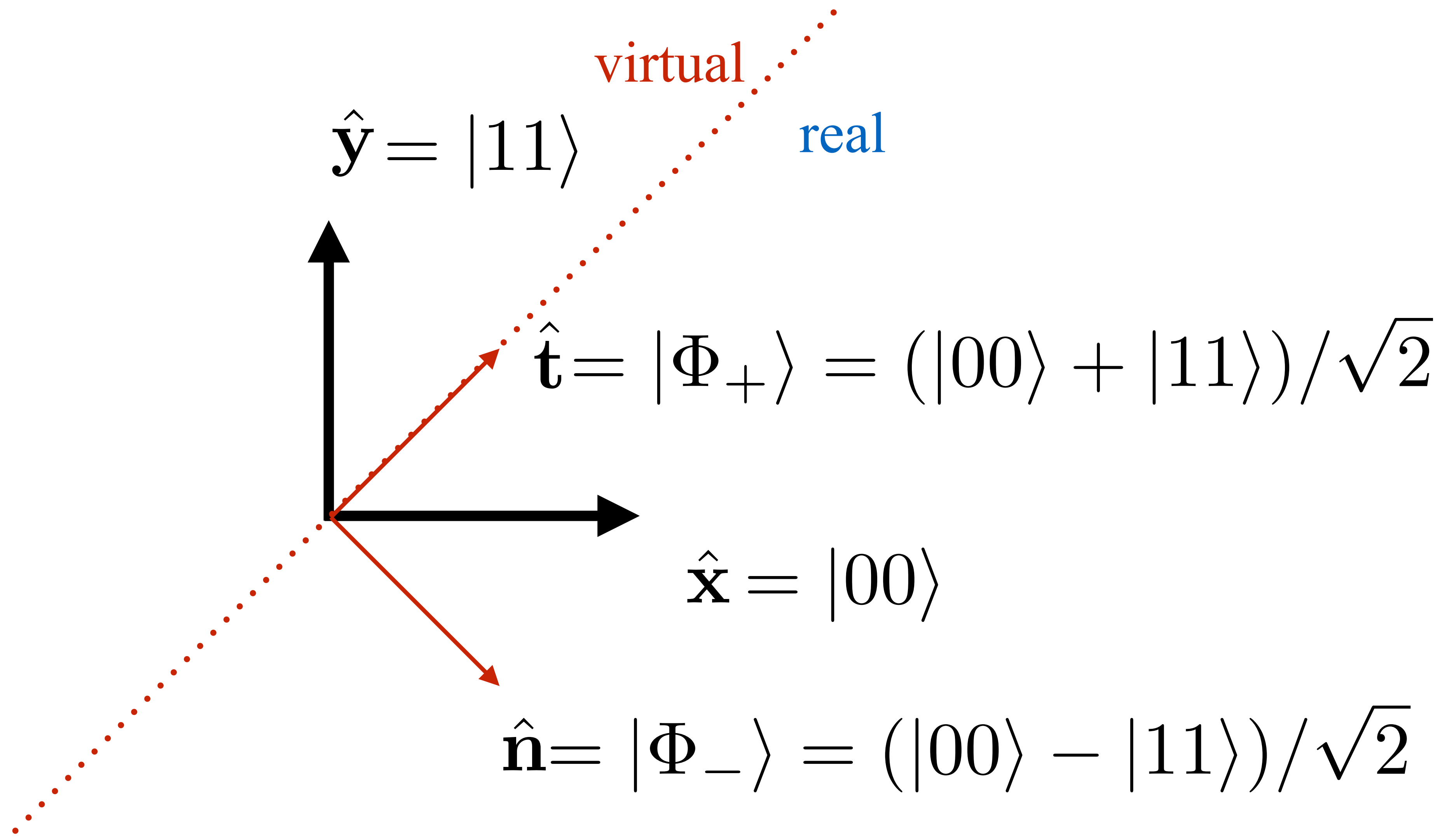

Supplement: Supplementary file 1 — Supplement [file 41598_2018_24489_MOESM1_ESM.pdf]
